# Evaluation of an Experience of Academic Happiness through Football at University

**DOI:** 10.3390/ijerph19116608

**Published:** 2022-05-28

**Authors:** David Almorza-Gomar, Rafael Ravina-Ripoll, Cristina Raluca Gh. Popescu, Araceli Galiano-Coronil

**Affiliations:** 1 Department of Statistic and Operative Research, University of Cadiz, 11003 Cadiz, Spain; david.almorza@uca.es; 2 Department of Business Organization and I.N.D.E.S.S., University of Cádiz, 11405 Jerez de la Frontera, Spain; rafael.ravina@uca.es; 3 Department of Business Administration, Faculty of Business and Administration, University of Bucharest, 030018 Bucharest, Romania; 4 Department of Economics and Economic Policy, Economics I Doctoral School, Faculty of Theoretical and Applied Economics, The Bucharest University of Economic Studies, 010374 Bucharest, Romania; 5 Marketing and Communication Department, Faculty of Social Sciences and Communication, University of Cadiz, 11406 Jerez de la Frontera, Spain; araceli.galiano@gm.uca.es

**Keywords:** football, physical education, academic happiness, descriptive analysis, university students, fair play, performance, competition, values, social responsibility, sustainability, education

## Abstract

The main objective of the university sport in Spain is the comprehensive training of the students. It sets out in the various state regulations in this respect. There is training in values within the comprehensive training that sporting activity should provide through Fair Play. This article aims to describe and evaluate an experience of training in values for the university students carried out by the Sports Department of the University of Cadiz, located in Cádiz, Andalusia, Spain. The methodology consisted of making selected changes to the game rules in football competitions. The experience has lasted ten years. The result of the experience has been very positive, obtaining, among other substantial achievements, a reduction of more than 75% in the percentage of cards (yellow and red) shown during matches and a reduction in referee cautions, a decrease in violent behavior, self-exclusion of players with violent behavior by the teams themselves, and an increase in fair play sporting behavior. Due to this experience, the Sports Department of the University of Cadiz has received numerous national and international awards. However, the leading award has been to take part positively, through sport, in the education and happiness of its students.

## 1. Introduction

In Spain, the Organic Law 4/2007 on Universities [1], which amends Law 6/2001 [2], states that “the practice of sport at university is part of the students’ training” (Article 90.1). Reference also make to university sport in the University Student Statute [1], which states that “sporting activity is a component of the integral education of the student” (Article 61.1).

Therefore, all the sports competitions, workshops, and related activities to sports, in general, that are being organized by different sports services or areas must pursue this training objective or have this comprehensive training component.

The Diagnostic Study of Spanish University Sport, carried out by Almorza et al. [3], shows that Spanish public and private universities promote sports activities in their facilities as a vital instrument for improving their students’ comprehensive training and happiness. Within the university sport, the activity with the most significant number of participants is represented by the football participants, considering as such the group formed by the modalities: 11-a-side football, 7-a-side football, and indoor football, which correspond to the same federation, the Royal Spanish Football Federation (R.F.E.F.). In this matter, the reference considers not only the overall aspects but also by taking into account the internal university competitions (C.I.U.), the regional university championships (C.A.U.), and the Spanish university championships (C.E.U.).

In each of the three types of competitions mentioned in the lines above, C.I.U., C.A.U., and C.E.U., the sports activity with the highest number of registered athletes is, in fact, football [3]. Perhaps this high participation is because, on a global level, it is the sport with the highest number of participants, the one with the most significant media impact, and it is also the most widespread sport. In this respect, Valencia-Candelija even associates football with a religious phenomenon [4]. The R.F.E.F. issued more than one million sports licenses in 2019, precisely 1,095,604 [5]. In continuation, a number very close to the total number of students enrolled in undergraduate studies reached during the 2018–2019 academic year in both public and private universities with on-site teaching in Spain, which reached the official figure of 1,102,678 [6].

Therefore, based on recent studies, football has also been included in teaching applications over time. García points out while referring to football, that: “parents and teachers do not always have content that may be of interest a priori to the youngest children, and therefore we face with a center of interest that can match the experience of the youngest children and encourage reasoning about more or less established prior knowledge” [7].

About the teaching applications that football has achieved, in this work, we are going to differentiate three models, as follows: (a) Those that use football as a pedagogical tool to explain concepts of specific subjects of a specific subject; (b) those that refer to football and, within it, deal with application contents dedicated to a transversal and cooperative teaching; and (c) those that use football with a comprehensive training function, without specifically academic contents.

In the first case, for those who use football as a didactic tool to explain concepts of specific topics of a particular subject, the example of [8] is relevant. They include examples of football for teaching statistical graphics. Likewise, the work of Carvalho et al. uses football to teach some issues within mathematics and geometry at the university level [9].

In the second model, those that refer to football and, within it, deal with application content dedicated to diverse teaching, the book by Sumpter is well-known. This particular book uses the word “schematics” to explain a wide variety of mathematical aspects of football [10]. Moreover, in the same vein, the work of Wesson uses mathematics, physics, marketing, and economics, seeking to explain issues related to football [11]. In like manner, Reilly y Williams included biology, medicine, and behavioral sciences, among others [12], to describe football, its characteristics, and its importance.

In the last case, those who use football with an integral and holistic educational function, i.e., without academic content, are, perhaps, the ones that are particularly attractive and represent, also, the subject of this paper. Football is considered a sport. As such, Prat et al. point out that: “sport is a privileged instrument for education in values” [13]. This third model, therefore, is a different kind of learning and consists of using only football as a psychological tool aimed at improving the integral formation of those who participate in the experience of playing this sport, not only as a process of socialization but also to enhance their health and individual happiness [14].

As a model, it may be the most widely used. However, the literature shows that few publications examine the pedagogical advantages of including football in their participatory learning process [15,16,17]. Among the precedents that can be mentioned in this respect, and making a selection of experiences, we can highlight, for example, the work of Márques et al. An analysis of the teaching of life skills through football is carried out [18].

On the other hand, there is the case of Font and Prat. They carried out an exciting project with the aim of “working on the values of civility, sportsmanship, and fair play, based on a confrontation between F.C. Barcelona and Real Madrid” [19], which is also referred to by Ginesta [20].

Jasso et al. conducted academic research on a strategy to promote values through football at the Frida Kahlo primary school in the city of Chihuahua in Mexico, highlighting that “the results obtained had a positive impact on students, teachers, and parents, resulting in attitudinal changes in the inside and outside the school” [21] (p. 97). A similar result was obtained by Jasso et al. [22] in the municipality of Garzón-Huila in Colombia. Another experience in primary education developed by Macías et al. using football as a psychological strategy for developing emotional intelligence [23].

A more recent and complete experience carried out by Almorza et al. [24] was developed simultaneously in three football teams of boys and girls aged between seven and thirteen of age. Six aspects of interest were analyzed from a comprehensive training program for male and female players in each of these teams. The aspects analyzed were: The academic training and study follow-up of the players; the assessment of the practice of fair play; the actions aimed at training in health, hygiene, and nutrition, as well as their compliance; the actions aimed at training the technical staff; the actions aimed at training the parents; and the actions aimed at issues of social responsibility. The results obtained were excellent.

Sánchez et al. analyzed prosocial behaviors in children and cadet football players, relating them to self-determination theory [25]. Prosocial behaviors are those actions that entail positive and happy consequences and aim to help, encourage, or benefit other people.

In an environment that, therefore, could appear to be conducive to the comprehensive education of students through football, one aspect that is of particular interest is how sportsmanship and fair play evolve in football according to the age of those who play it.

Cruz et al. show a statistically significant loss of these values provided by fair play at the youth age, usually the age that coincides with university entrance for students who follow this educational pathway [26]. From the University’s point of view, students who play football at youth age constitute the primary cohort of new students entering each academic year.

However, there are few experiences focused on fair play at university age. It is worth highlighting the one carried out by Palou et al., who conducted a study on appropriate play behavior in physical education teacher training students at the University of the Balearic Islands (Spain) [27]. Perhaps one of the reasons for this phenomenon currently lies in the fact that the didactics of higher education institutions in Spain are oriented toward promoting instruction that cultivates the academic happiness of future university graduates [28].

Nevertheless, solid ethical values represent the key to all successful activities and ventures, especially when targeting long-term performance and when willing for long-term individual and group development [29]. Moreover, solid ethical values should implicate continuously acting in the spirit of social responsibility, fair play, constructive performance, competition based on solid values, and the highest moral principles in terms of personal and professional activities [30].

Hence, the need to implement learning models within University 5.0 that imbue the practice of football and the pursuit of happiness of university students as didactic tools to promote teamwork, interpersonal skills, tolerance and equality [31].

On this premise, this paper aims to show the formative experience in the field of fair play carried out by the University of Cadiz (Spain) during the first years of the 21st century. In this regard, it should note that the University of Cadiz had nearly twenty-two thousand students enrolled in the 2018–2019 academic year, precisely 21.860 students according to its Social Responsibility and Sustainability and Responsibility Report [32].

## 2. Materials and Methods

To begin this experience, and first, and from the Sports Area of the University of Cadiz, a Decalogue of Fair Play at the University of Cadiz was published, developed in Terol [33]. All nine Andalusian public universities signed a Code of Ethics for Andalusian University Sport, whose prologue states that “Andalusian universities consider physical activity and sports practice as a fundamental part of the training of their students”, the full content of this code being available at the University of Cadiz [34].

As reported in Almorza et al., this Code of Ethics affected, at the time of its signing, around 250,000 students (241,346; 44.73% male and 55.27% female) which, when completed with the sum of the teaching and non-teaching staff of each of the nine universities, covers a figure of around 270,000 people (more specifically, 266,296 people; 45.85% male and 54.15% female) [35].

Once the objective was established, a Decalogue developed and, as a result of the Decalogue, a standard sports Code of Ethics for the nine Andalusian universities had been created and signed; the next step was to transfer this fair play approach to the rules of the game in football, also including football 7-a-side and futsal, which word as follows:

In the case of 11-a-side football (7-a-side football or futsal) matches in a regular league, 3 points are awarded to the winning team at the end of each match and 0 points to the losing team. In the event of a tie, 1 point is awarded to each team. In addition, it establishes that each team may:

- To add one point, the teams that have reached the end of the match without having any player sanctioned with a card and without the referee have made a negative note on the score sheet.
- Subtract one point: to teams that have been penalized with at least three yellow cards during the match, taking into account that for every three additional yellow cards, one more point will be subtracted; and also, if any team has been penalized with red cards for severe unsportsmanlike conduct (insults, threats, aggressions), taking into account that for every additional red card one more point will be subtracted.

It notes that two red cards for two cautions, except those resulting in severe unsporting behavior, and direct red cards for handling an apparent goal-scoring opportunity will be considered two yellow cards for additional points. Table 1 includes a synthesis of the explanation of these rules:

For 11-a-side football (7-a-side football or futsal) matches that form part of a final or knockout phase, the number of goals is taken into account instead of acting on the number of points per match. In this way, it establishes that each team will be able to add goals to its scoreboard according to the behavior of the opposing team as specified below:

- Adding a goal: if the opposing team shows three yellow cards or more; if the opposing team is shown a straight red card (except in the case of a double caution or for handling an apparent goal-scoring opportunity or if they are penalized with a penalty kick and sent off).
- Add two goals: if the opposing team commits severe unsporting behavior, recorded on the score sheet.

In the event of a tie, each team will take three penalty kicks, and if the tie persists, the kicks will continue until the tie breaks. It should be noted that any unsporting attitude of any team during the kicks will result in the loss of the tie. Table 2 presents the explanation of these rules:

It is necessary to repeat that university sport is not aimed at the competition, nor is it aimed at preparing professional football players. As specified at the beginning, the objective of university sport is the necessary training and academic happiness of the athlete—namely, training in values that are above and beyond the idea of the usual sporting competition.

For this reason, the rules established in this experience for university football competitions may clash with the usual ones. However, they correspond to a change of mentality, to a change in the object of work, that is, to the fact that a football match is part of the integral formation of the person who plays it, who is also a person studying at the University.

Some references to this experience, in addition to Terol [33], have been presented by Prat et al. [36], who stress that introducing changes in the context of the internal league and modifying some rules “can be a good way to increase participation and favor fair play proposals”; and, also, Lamoneda and Huertas, who indicates that it has been an “effective initiative” [37].

An introductory preview of the first four years of the experience can be found in Almorza and Prada. The work is completed exhaustively by carrying out the final evaluation [38]. The experience has been prolonged over time and, at present, for various reasons. Unfortunately, due to the pandemic caused by COVID-19, it has been interrupted. In any case, it is the most continuous and prolonged experience of comprehensive training through football at the university level that we have found.

This paper evaluates ten years of experience, from the 2008–2009 academic year to the 2017–2018 academic year, inclusive and each year’s effects since its implementation at the University of Cadiz. As a starting point, the data obtained in the academic year 2007–2008, the year before this experience, are taken as a reference point.

As a tool to evaluate the experience we are going to considerer, for each year the following key issues: number of matches played, number of yellow, red, and total of cards shown, percentage of decrease in yellow, red, and total cards, the ratio of yellow, red, and total cards shown per match and percentage of decrease in the ratio of yellow, red, and total cards.

In this study, we considered all the participants of all the matches played during these ten years and the year before as a reference. This study carries out in 2925 matches.

## 3. Results

During the academic year 2007–2008, 439 11-a-side football, 7-a-side football and indoor football matches were played at the University of Cadiz in official competitions regulated by the Sports Department, i.e., within an official competition established with referees appointed by the Cadiz Delegation of the Royal Andalusian Football Federation. During these matches, 815 cards show, 704 were yellow cards, and 111 were red cards. To translate this into comparable units irrespective of the number of matches played, this is 1.86 cards per match, divided into 1.61 yellow cards per match and 0.25 red cards per match.

Ten years later, in the 2017–2018 academic year, 110 11-a-side football, 7-a-side football and futsal matches were played under the same conditions. A total of 45 cards show, of which 39 were yellow cards and six red cards. That is 0.41 cards per match, with 0.36 yellow cards per match and 0.05 red cards per match.

It means that, after ten years of training experience, the number of cards shown per match played has reduced by 77.96%, the number of yellow cards shown by 77.89%, and the number of red cards by 78.43%. These represent significant percentages that speak of the improvement in the players’ behavior on the pitch.

This impressive balance shown in Table 3 and Table 4 presents the data on matches played, the total number of yellow and red cards shown (Table 1), and the percentages of relegation compared to the previous season. These are overall values.

Table 3 shows the evolution of the number of cards (yellow and red) shown in the four academic years analyzed and the percentage decrease concerning the previous season. In this particular matter, it should be noted that the number of matches fluctuated from one academic year to another, depending on the program elaborated by the University of Cadiz concerning the official competitions regulated by the Sports Department. In continuation, it should highlight that from the very moment in which the selected changes to the rules of the game in football are made, it can be noticed that the participants in the university competitions were more determined and more motivated to focus on fair play approach to the rules of the game in football. In this matter, at the University of Cadiz, football started as being regarded by the students as a sport that has the power to be an essential part of their necessary training and academic happiness as athletes, which led to the importance of the entire process represented by training in values that are above and beyond the idea of the usual sporting competition and performance. For instance, the values displayed in Table 3 point out the fact that in the academic year 2014–2015, during the 230 matches, a total number of 173 cards were shown, signifying several 149 yellow cards shown and several 24 red cards shown, which reflects, in essence, a 78.77% decrease in terms of cards shown. In addition, another example can be represented by the academic year 2015–2016, where during the 171 matches, a total number of 119 cards were shown, signifying several 103 yellow cards shown and several 15 red cards shown, which reflects, in essence, an 85,40% decrease in terms of cards shown. In continuation, the positive evolution of the values belonging to the academic year 2016–2017, where during the 154 matches, a total number of 55 cards shown, signifying several 50 yellow cards shown and several five red cards shown, which reflects, in essence, a 93.25% decrease in terms of cards shown. Also, it ought to be stressed that the results bellowing to very next academic year, 2017–2018, proved to be highly rewarding in terms of the efforts put during the competitions that took place, since, during the 110 matches, only a total number of 45 cards shown, representing several 39 yellow cards shown and several six red cards shown, which reflects, in essence, a 94.48% decrease in terms of cards shown.

Table 4 shows the values relating to the number of matches played in each of the academic years of application of this experience and the percentage improvement obtained.

**Figure 1:**
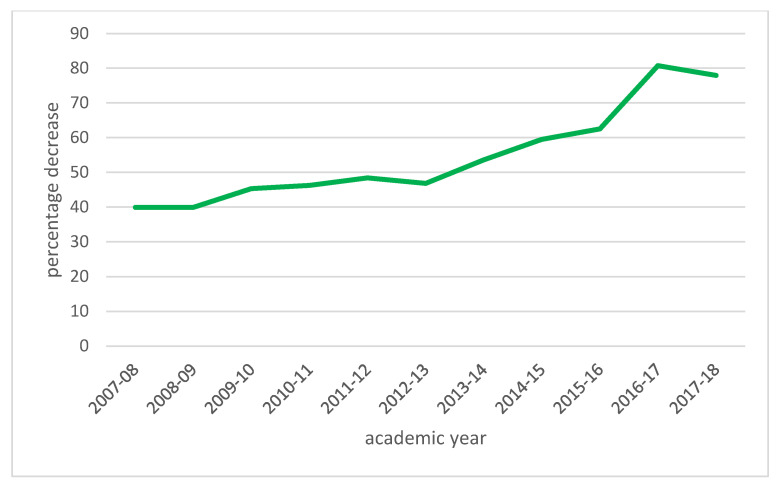
Total cards percentage decrease during the ten years. Source: Own elaboration.

Table 4 presents the evolution of the average number of cards per match in the four academic years analyzed and the percentage decrease compared to the previous season. By closely analyzing this table, it can prompt that the new rules that have been established in this experience for university football competitions—although, in some ways, different from the usual ones, implicate a severe change in terms of individuals’ mentality toward sports, completion, and performance at a university level, thus reflecting an effective change in the object of work, essentially capturing the main idea that all football matches should assimilate with the part of the integral formation of the persons who play these games, who are, also, the persons who study at the University. Figure 1 represents the evolution during these years.

To appreciate this decrease, we introduce three ratios in the total matches played: ratio of yellow cards; ratio of red cards and ratio of total cards (by addition of yellow and red cards for each academic year. These ratios are shown in Table 5.

**Figure 2:**
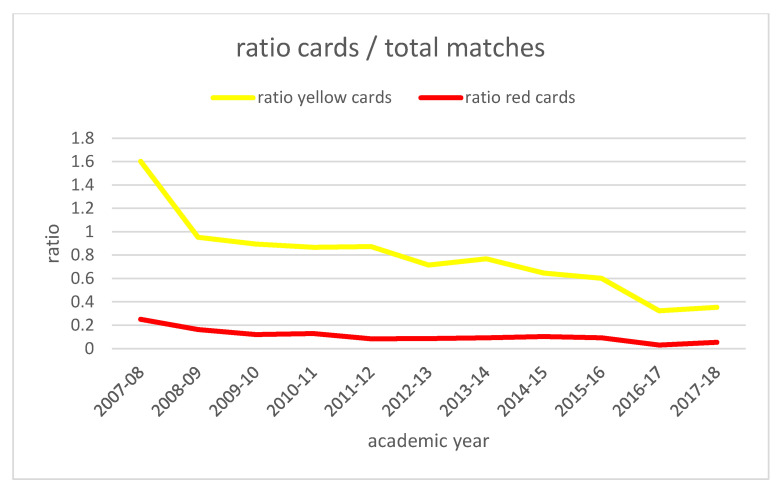
Evolution of ratio of yellow cards and ratio of red cards through the academic years. Source: Own elaboration.

**Figure 3:**
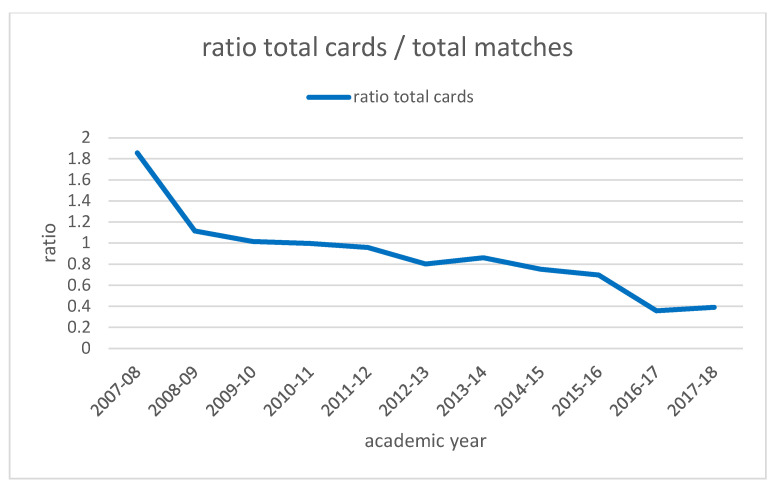
Evolution of ratio of total cards through the academic years. Source: Own elaboration.

**Figure 4:**
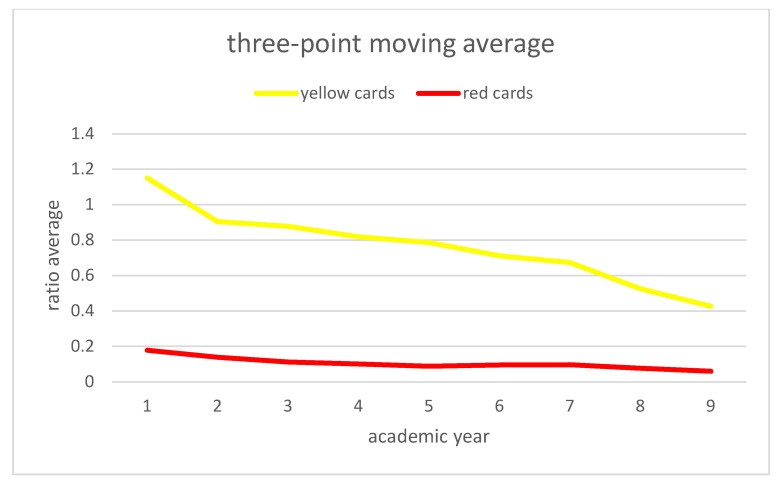
Three point moving average for yellow and red cards. Source: Own elaboration.

**Figure 5:**
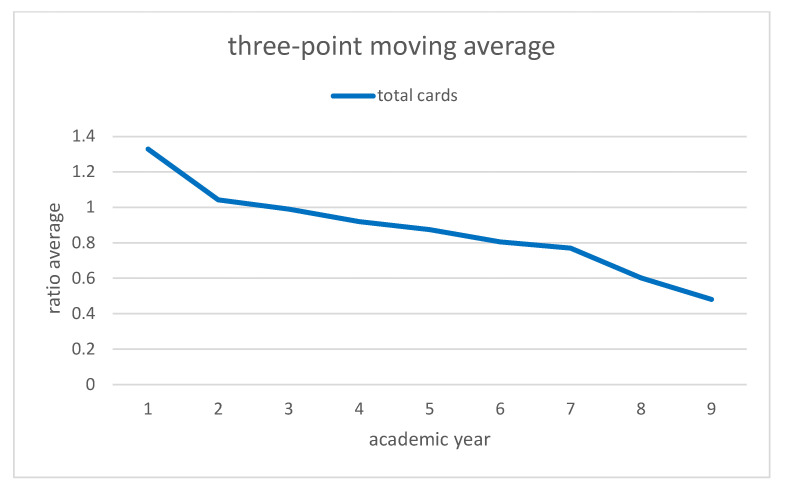
Three point moving average for total cards. Source: Own elaboration.

Figure 2 and Figure 3 represent the evolution of these ratios through the academic years. As they are time series, and in order to reduce the effects of random variation, we applied the three point moving average as it is shown in Figure 4 and Figure 5.

In this way we obtain both strictly decreasing series in the ratio of yellow cards and in the ratio of total cards. Moreover, a decreasing series in the ratio of red cards was seen. For this reason, also with the percentage of decrease, we can say that these new rules are effective and improve the fair play.

## 4. Conclusions

The conclusions highlighted by the players, female players, people acting as members of the technical staff, referees, and female referees who have acted by designation of the Cadiz Delegation of the Royal Andalusian Football Federation [39], as well as the technical staff and the administration and services staff who are part of the Sports Area of the University of Cadiz, are the following:

- There is an apparent decrease in violence during matches, measured in the number of yellow and red cards shown per match during the matches played.
- There is a decrease in the number of annotations made on the score sheet by the competition committee for unsporting behavior.
- We can observe an exclusion that we could call endogenous, produced by social reprobation within the team itself.
- An internalization of fair play and sporting behavior. It means that fair play is considered normal for the participants in football matches. It is in line with the ethical principles of sport and, therefore, football.

Following this line of pedagogical action can serve to include academic happiness in the curricula of Spanish universities, provided that it harmoniously integrates with the social, affective, and personal development of future graduates and postgraduates. Perhaps this may reduce, on the one hand, the high percentage of students who leave their university system without having graduated [40]. Moreover, on the other hand, encouraging the creation of happiness chairs as in American universities, whose teaching is characterized, among other things, by having teachers with the role of coach, that is, with the ability to motivate their students, energize community spaces, promote collaborative work, etc. [41]. In sum, with the above, future university education policies in the post-COVID-19 era should aim to cultivate the academic happiness of their students through the trinomial of cognitive motivation, subjective well-being, and sport. It achieves in educational systems that are mindful of the words of the famous Enlightenment philosopher, “how wonderful it is to imagine that human nature can develop better and better because of education (…). These open us to the prospect of a happier human species”.

In continuation, it should be underlined that the post-COVID-19 era ought to focus more on the well-being and health of individuals in order to help individuals grow and develop at a superior level, thus increasing people’s level of satisfaction and happiness when it comes to analyzing all aspects of their lives [42], also while noting the importance of the Sustainable Development Goals (SDGs).

By way of conclusion, it should be pointed out that the training experience described in the previous pages has been rewarded with many recognitions and distinctions during this time. Between 2009 and 2010 it was awarded the Aula Abierta Prize by the Faculty of Communication Sciences of the University of Seville (Spain); the Prize for the Best Sports Initiative by Gaceta Universitaria; the Ciudad de Cádiz Sports Prize 2010 (awarded by the City Council of Cádiz); the Special Mention at the Provincial Sports Gala of Cádiz (awarded by the Provincial Council of Cádiz); the Andalusia Sports Prize (awarded by the Provincial Council of Cádiz); the Premio Andalucía de los Deportes (awarded by the Junta de Andalucía); the Placa de Bronce de la Real Orden del Mérito Deportivo (awarded by the Consejo Superior de Deportes) (University of Cádiz, 2011); the Premio Nacional del Deporte Trofeo Joaquín Blume (also awarded by the Consejo Superior de Deportes) (Consejo Superior de Deportes, 2009: 27); and, also, the World Fair Play Diploma (recognition awarded by the International Fair Play Committee founded by UNESCO) (University of Cadiz, 2011) [43,44]. It should be noted that owing to this experience, the University of Cadiz has the honor of becoming the first University to obtain the World Fair Play Diploma.

It should be highlighted that this current paper targeting the evaluation of an experience of academic happiness through football at university, focuses successfully on valuable key concepts, such as, football; physical education; academic happiness; university students; fair play; performance; competition; values; social responsibility; and education, using as method the descriptive analysis. Moreover, the results obtained in this paper come to support the importance of sports and of sports events, not solely at the level of universities, but at a larger scale, since “global events, (…), cultural festivals or world expositions, have long been seen as opportunities to re-invigorate local growth and optimize local assets” [45] (p. 3), especially in the COVID-19 pandemic, and in the context in which the organizers of such important events “have sought to leverage long-term infrastructure investments, boost tourism and trade, create jobs and promote community engagement” [45] (p. 3).

Furthermore, specialists worldwide have shown lately a great concern toward individuals’ health, in terms of “immunity and mental well-being”, especially in the context in which “in recent history, health has been driven by corporations rather than citizens” [46] (p. 5), but “as the world moves towards longevity, awareness of immunity and mental well-being as global common goods is imperative for a healthier and a happier society” [46] (p. 5). Thus, sports performed at the youth age, under fair play conditions, and in terms of high social responsibility standards, have the tremendous power to “raise awareness of the importance of immunity and well-being” at the level of all individuals, and, in the same time, “(…) would tie in perfectly with environmental initiatives on clean/quality air” promoted and supported by the United Nations (U.N.) [46] (p. 5).

This current study has several limitations, which summarizes in the lines below:

- The first limitation that we can point out is the country chosen for analysis and the number of subjects chosen for analysis. In this case, the aim was to describe and evaluate experience of training in values for the university students carried out by the Sports Department of the University of Cadiz, located in Cádiz, Andalusia, Spain. Nevertheless, it is our firm opinion that if similar changes were decided and adopted at other universities, the students would have increased and added clear benefits from the new experience, since they would have the opportunity to concentrate more on the idea of playing a positive game, while focusing on interpersonal relationships, building constructive strategies, taking care of their health and centering on happiness, mindfulness and well-being approaches. In continuation, the benefits would be immense by taking physical education as an opportunity to improve personal skills in terms of sports strategies, ways of improving performance, and methods to compete with oneself rather than with other colleagues. The results would turn out to be highly promising no matter of the University or group of subjects selected for the analysis.
- The second limitation that we can address refers to the fact that the new rules which so successfully applied by the Sports Department of the University of Cadiz, located in the province of Cádiz, Andalusia, Spain—thus, at the university level, may not prove to be fit to the general rules and regulations that are used outside universities, since these rules established in this experience for university football competitions may clash in some cases with the usual ones. However, we do believe that by trying to change professional sports players’ general mentality, in terms of concentrating more on constructive, positive, and happy situations and strategies, by using mindfulness and well-being approaches, an apparent decrease in the level of violence during matches, measured in terms of the number of yellow and red cards shown per match during the matches played, could be encountered in these cases as well.

## Figures and Tables

**Table 1:** Rules in the regular league.

Regular league: 11-a-side football; 7-a-side football; futsal.
**Result of the match:** Winning team: 3 points. Losing team: 0 points. Tie: 1 point to each team.
**Fair play. Additional points** Team without cards: 1 point. Team with 3 yellow cards: −1point. Every additional 3 yellow cards: −1point. Every red card: −1 point.

Source: Own elaboration.

**Table 2:** Rules in final of knockout phase.

Final of knockout phase: 11-a-side football; 7-a-side football; futsal.
**Result of the match:** Number of goals.
**Fair play. Additional points:** Opposing team with 3 yellow cards or more: 1 goal. Opposing team with cards (with exceptions): 1 goal. Opposing team with serious unsporting behaviour: 2 goals.

Source: Own elaboration.

**Table 3:** Evolution of the number of cards shown in the ten academic years analyzed, and percentage decrease concerning the previous season.

Academic Year	Matches Played	Yellow Cards	% Decrease	Red Cards	% Decrease	Total Cards	% Total
2007–2008	439	704	-	111	-	815	-
2008–2009	313	298	57.67	51	54.05	349	57.18
2009–2010	323	289	58.95	39	64.86	328	59.75
2010–2011	337	292	58.52	44	6036	336	58.77
2011–2012	308	269	61.79	26	76.58	295	63.80
2012–2013	302	216	69.32	26	76.58	242	70.31
2013–2014	238	183	74.01	22	80.18	205	74.85
2014–2015	230	149	78.84	24	78.38	173	78.77
2015–2016	171	103	85.37	16	85.59	119	85.40
2016–2017	154	50	92.90	5	95.50	55	93.25
2017–2018	110	39	94.46	6	94.59	45	94.48

Source: Own elaboration.

**Table 4:** Evolution of the average number of cards shown per match in the ten academic years analyzed, and percentage decrease compared to the previous season.

Academic Year	Yellow Cards per Match	% Decrease	Red Cards per Match	% Decrease	Total Cards per Match	% Total
2007–2008	1.60	-	0.25	-	1.86	-
2008–2009	0.95	40.63	0.16	35.56	1.12	39.94
2009–2010	0.89	44.21	0.12	52.25	1.02	45.30
2010–2011	0.87	45.97	0.13	48.36	1.00	46.29
2011–2012	0.87	45.54	0.08	66.61	0.96	48.41
2012–2013	0.72	55.40	0.09	65.95	0.80	56.84
2013–2014	0.77	52.05	0.09	63.44	0.86	53.60
2014–2015	0.65	59.60	0.10	58.73	0.75	59.48
2015–2016	0.60	62.44	0.09	62.99	0.70	62.51
2016–2017	0.32	79.75	0.03	87.16	0.36	80.76
2017–2018	0.35	77.89	0.05	78.43	0.41	77.96

Own elaboration.

**Table 5:** Evolution of the ratios of cards shown in the ten academic years analyzed, and ratios concerning the previous season.

Academic Year	Matches Played	Yellow Cards	Ratio of Yellow Cards (SD)	Red Cards	Ratio of Red Cards (SD)	Total Cards	Ratio of Total Cards (SD)
2007–2008	439	704	1.604 (33.6)	111	0.253 (5.3)	815	1.856 (38.9)
2008–2009	313	298	0.952 (16.8)	51	0.163 (2.9)	349	1.115 (19.7)
2009–2010	323	289	0.895 (16.1)	39	0.121 (2.2)	328	1.015 (18.2)
2010–2011	337	292	0.866 (15.9)	44	0.131 (2.4)	336	0.997 (18.3)
2011–2012	308	269	0.873 (15.3)	26	0.084 (1.5)	295	0.958 (16.8)
2012–2013	302	216	0.715 (12.4)	26	0.086 (1.5)	242	0.801 (13.9)
2013–2014	238	183	0.769 (11.8)	22	0.092 (1.4)	205	0.861 (13.3)
2014–2015	230	149	0.648 (9.8)	24	0.104 (1.6)	173	0.752 (11.4)
2015–2016	171	103	0.602 (7.8)	16	0.094 (1.2)	119	0.696 (9.1)
2016–2017	154	50	0.325 (4.0)	5	0.032 (0.4)	55	0.357 (4.4)
2017–2018	110	39	0.354 (3.7)	6	0.055 (0.6)	45	0.391 (4.1)

Source: Own elaboration.

## Data Availability

Not applicable.
